# Comparatio inter sectionem Cæsaream et dissectionem cartilaginis et ligamentorum pubis in partu, ob pelvis angustiam, impossibili, succipiendos

**Published:** 1781-07

**Authors:** 


[ *To be continued.* ]

---

III. *Comparatio inter ſectionem Cæſaream et diſſectionem cartilaginis et ligamentorum pubis in partu, ob pelvis anguſtiam, impoſſibili, ſuccipiendos, Auctore* C. C. Siebold, M. D. *Anat. Chirurg. et artis obſtetriciæ Prof. pub. ord.* 4to. Wirteburgi, 1779. p. 72.

THE author begins this candid and judicious performance, with a hiſtory of the Cæſarean operation, from the time of Rouſſet, one of the earlieſt writers on the ſubject, to the preſent day; after which he gives a ſhort account of a caſe in which he himſelf had occaſion to perform it. The patient, we are told, was ricketty and deformed. Dr. Siebold had aſſiſted her in a former pregnancy, when the head of the child was opened, and the delivery accompliſhed with great difficulty. In this ſecond labour, the ſtate of the patient, and the apparent impoſſibility of her being relieved by any other method, induced him to perform the Cæſarean operation.

He

He began by making an incision through the integuments on the right side of the abdomen, this being more distended than the left. Unfortunately, in cutting into the uterus he wounded the placenta, which occasioned a profuse hæmorrhage. The child was extracted alive by the feet, and the uterus immediately contracted; after which the hæmorrhage lessened, and the placenta being detached, was extracted without difficulty. The omentum protruded through the upper part of the wound of the integuments, but was easily reduced, after which the lips of the wound were kept together by slips of sticking plaster.

The night succeeding the operation the patient was attacked with a violent vomiting, by which the lips of the wound were burst open, the omentum and small intestines pressed through it, and the hæmorrhage excited afresh. The day following the patient had symptoms of fever, complained of pains in her belly, and had frequent inclination to vomit. On the fourth day a dark brown ichor began to issue from the wound; on the fifth the vomiting ceased; and on the eighth the patient died.

Upon dissection, the intestines, omentum, and mesentery were found in several places adhering together in an inflamed and gangrenous state; and in the neighbourhood of the *inteſtinum cæcum* and bladder was a conſiderable quantity of black corrupted blood, partly in a coagulated, partly in a diſſolved ſtate.

The child, ſaved by the operation, was afflicted three weeks after its birth with ulcerations over its whole body, and ſoon died. The nurſe who ſuckled it found her nipples and breaſts affected in a ſimilar manner. Theſe appearances our author attributes to the lues venerea with which both the mother and child were probably infected.

Dr. Siebold next proceeds to the hiſtory of the new operation for dividing the ſymphyſis pubis in caſes of difficult parturition, the invention of which is due to M. Sigault of Paris. This operation has now been ſucceſsfully performed by ſeveral practitioners in different parts of Europe, and amongſt others by our author, from whoſe account of it we ſhall extract the following particulars:

When he had ſeparated the oſſa pubis with a ſaw, they receded from each other about three lines, or in other words, three tenths of an inch, but in order to increaſe the ſeparation to ſixteen lines, the diſtance that was required, ſo much violence was obliged to be employed, that a total laceration of the bladder and vagina was dreaded, and and the turning of the child proved ſo tedious and difficult, that our author's ſtrength being exhauſted, an aſſiſtant was obliged to complete it. If the child had been alive, which was not the caſe, he thinks it would probably have died during the operation.

After her delivery the patient had an incontinence of urine, but the lochia flowed as uſual. On the fifth day ſhe complained of a violent pain in the region of the ſacrum, which continued two days, and then went off. On the 10th the urine began to flow through the wound. On the 11th the patient was able to walk about her chamber without difficulty, although the oſſa pubis were certainly not concreted, and ſloughs were to be perceived in the wound, from the appearance of which Dr. Siebold ſuppoſes that a mortification had begun to take place about the neck of the bladder. The day following, upon examination, the bones of the pubis were found to be unequal and carious, and a crackling noiſe could be diſtinctly heard whenever ſhe walked.

On the 32d day a piece of bone exfoliated, and the ſame thing happened ſeveral times afterwards during the courſe of the cure. In about ſeven days after this the patient began to void her urine through the natural channel, but ſome of it ſtill continued to paſs now and then through the wound. On the 39th day after the operation ſhe was able to walk up and down ſtairs without difficulty, although the bones of the pubis were not perfectly united till the 52d day, when ſhe went about her uſual buſineſs, having no other complaint than a ſmall fiſtulous opening which was left to nature.

In July 1779, a year after the operation, our author ſaw her again. The fiſtula was then healed, after ſeveral exfoliations had taken place, and ſhe was in perfect health. By introducing his finger into the vagina he was able plainly to diſtinguiſh the callus between the two bones of the pubis.

After relating this caſe, Dr. Siebold proceeds to the comparative inquiry mentioned in the title of his work, and begins with conſidering the difficulties that attend the Cæſarean operation: of theſe, he obſerves, the principal are the idea of horror that is naturally annexed to it; the uncertainty in ſome caſes whether the child is alive or dead; the danger of wounding the placenta, by the inciſion into the uterus, and the dreadful hæmorrhage that enſues from thence; the protruſion of the inteſtines and omentum through the wound; the adheſion of the inteſtines to the wound wound of the uterus, in conſequence of inflammation, as M. Louis of Paris once ſaw happen; the want of proper means to bring the lips of the external wound together, ſlips of ſticking plaſter not being ſufficient for this purpoſe, and the uniting them by means of a needle and thread never failing to increaſe the irritation, pain, and inflammation; the violent vomiting that uſually follows the operation, with all its dangerous conſequences; and laſtly, the danger of a hernia after a complete cure. Our author next directs his views to *ſynchondrotomy*, or the ſection of the ſymphyſis pubis, an operation, he obſerves, which is neither ſo dangerous nor ſo painful as the one juſt now ſpoken of; but he thinks that on this ſubject the principal queſtion is, whether it is really ſo advantageous as its advocates have taught us to imagine. In diſcuſſing this point our author remarks, that by a moderate ſeparation of the bones of the pubis from each other, the diameter of the pelvis is but very little augmented; and that if much violence is employed for this purpoſe, there will be great danger of lacerating the ligaments of the pelvis, and of courſe bringing on a fatal ſuppuration. He obſerves likewiſe, that much force cannot be employed without injuring the bladder, and its

 ſur-

ſurrounding cellular membrane by the extenſion. If the narrowneſs of the pelvis is owing to an exoſtoſis the operation will, in his opinion, be fruitleſs, and he thinks it will be equally ineffectual if the ligamentous fibres at the ſides of the os ſacrum are oſſified; inſtances both of the one and the other of theſe caſes have occurred to him in his diſſections. Upon the whole he is of opinion that this operation will be found uſeful only in caſes where the head of the child is not of extraordinary bulk, and where the diameter of the pelvis is not leſs than three inches.

